# Supplementary material for: Mechanisms governing the pioneering and redistribution capabilities of the non-classical pioneer PU.1
Source: Nat Commun. 2020 Jan 21;11:402. doi: 10.1038/s41467-019-13960-2 (PMC6972792; doi:10.1038/s41467-019-13960-2)
Supplement: Supplementary file 7 — Source data [file 41467_2019_13960_MOESM7_ESM.zip › Source_Data/FigureS3/FigureS3H_MotifScanOutput/homerResults/motif7.similar.html]

motif7

## Information for motif7

T
A
G
C
G
A
C
T
A
C
G
T
G
C
A
T
T
C
A
G
C
T
G
A
C
G
A
T
T
A
C
G
C
A
G
T
C
T
A
G
C
T
A
G
  
Reverse Opposite:  

G
A
T
C
G
A
T
C
G
T
C
A
A
T
G
C
C
G
T
A
G
A
C
T
A
G
T
C
C
G
T
A
T
G
C
A
C
T
G
A
A
T
C
G
  

|  |  |
| --- | --- |
| p-value: | 1e-120 |
| log p-value: | -2.770e+02 |
| Information Content per bp: | 1.519 |
| Number of Target Sequences with motif | 622.0 |
| Percentage of Target Sequences with motif | 16.09% |
| Number of Background Sequences with motif | 2318.3 |
| Percentage of Background Sequences with motif | 5.17% |
| Average Position of motif in Targets | 256.3 +/- 237.3bp |
| Average Position of motif in Background | 209.0 +/- 148.0bp |
| Strand Bias (log2 ratio + to - strand density) | 0.1 |
| Multiplicity (# of sites on avg that occur together) | 1.07 |
| Motif File: | file (matrix) reverse opposite |

### Similar de novo motifs found

|  |  |  |  |  |  |  |  |
| --- | --- | --- | --- | --- | --- | --- | --- |
| Rank | Match Score | Redundant Motif | P-value | log P-value | % of Targets | % of Background | Motif file |
| 1 | 0.942 | G C A T G A C T C G A T T A C G C G T A C G A T A T C G C G A T C A T G A C T G | 1e-114 | -264.496955 | 9.88% | 2.11% | motif file (matrix) |
| 2 | 0.943 | A G T C G A T C G A C T A C G T C G A T A T C G C G T A C G A T A T C G C G A T C T A G C T A G | 1e-101 | -233.904271 | 7.79% | 1.45% | motif file (matrix) |
| 3 | 0.916 | G T A C G T A C G C T A T A G C G C T A G C A T A T G C G C T A T G C A C T G A C A T G T A C G T C G A | 1e-98 | -227.856390 | 11.54% | 3.28% | motif file (matrix) |
| 4 | 0.890 | T A G C A G C T A C G T G C A T A T C G G T C A C G A T A T C G G A C T | 1e-74 | -171.005998 | 28.84% | 16.45% | motif file (matrix) |
| 5 | 0.792 | A G T C G A T C A G C T A G C T C G A T A T C G C G T A C G A T A T G C G C A T | 1e-64 | -149.550054 | 18.18% | 8.91% | motif file (matrix) |
| 6 | 0.759 | C A G T A T G C G T A C A C G T A G C T A C G T A T C G G C T A C G A T T A G C A C G T A C T G | 1e-35 | -82.018898 | 7.66% | 3.23% | motif file (matrix) |
